# EUS point shear-wave elastography: A novel approach for noninvasive liver fibrosis assessment

**DOI:** 10.1097/eus.0000000000000195

**Published:** 2026-05-11

**Authors:** Bogdan Miutescu, Adrian Burdan, Eyad Gadour, Antonio Facciorusso, Mohammed AlQahtani, Iulia Ratiu, Camelia Nica, Ana Maria Ghiuchici, Mirela Danila, Roxana Sirli, Alina Popescu

**Affiliations:** 1 Division of Gastroenterology and Hepatology, Department of Internal Medicine II, “Victor Babes” University of Medicine and Pharmacy, Timisoara, Romania; 2 Advanced Regional Research Center in Gastroenterology and Hepatology, “Victor Babes” University of Medicine and Pharmacy, Timisoara, Romania; 3 Doctoral School, “Victor Babes” University of Medicine and Pharmacy, Timisoara, Romania; 4 Multi-Organ Transplant Centre of Excellence, Liver Transplantation Unit, King Fahad Specialist Hospital, Dammam, Saudi Arabia; 5 Department of Medicine, Faculty of Medicine, Zamzam University College, Khartoum, Sudan; 6 Department of Experimental Medicine, Gastroenterology Unit, Università del Salento, Lecce, Italy.

**Keywords:** elastography, shear wave, EUS, liver cirrhosis, fibrosis, prospective studies

## Abstract

**Background and Objective:**

EUS point shear‑wave elastography (EUS‑pSWE) permits near‑field, intraluminal assessment of liver stiffness.

We examined its concordance with vibration-controlled transient elastography (VCTE) and its diagnostic accuracy for advanced fibrosis (F ≥ 3) in an expanded mixed cohort.

**Methods:**

One hundred twenty adults (57% men; mean age: 61 ± 10 years; body mass index: 28.9 ± 4.0) undergoing routine endoscopic ultrasound were prospectively enrolled. VCTE stratified fibrosis as F0–1 (<7.0 kPa, *n* = 96), F2 (7–9.4 kPa, *n* = 11) and F3‑4 (≥9.5 kPa, *n* = 13). In all the patients, 10 EUS‑pSWE measurements were performed in each lobe, and the liver stiffness result was the median value of the 10 measurements. Correlation (Pearson’s *r*), Bland–Altman agreement, and diagnostic indices for F ≥3 were calculated.

**Results:**

Median EUS‑pSWE increased with fibrosis (left lobe: 5.0 ± 0.9, 7.8 ± 1.0, 15.1 ± 3.4 kPa for F0–1, F2, F3–4; *P* < 0.001). EUS-pSWE correlated strongly with VCTE (*r* = 0.89, left; *r* = 0.88, right; *P* < 0.0001). AUROC for detecting F ≥3 reached 0.91 (95% confidence interval: 0.85–0.96) for the left and 0.89 (0.83–0.95) for the right lobe. A 10.2 kPa cutoff value for F3 afforded 85% sensitivity, 84% specificity, positive predictive value 72%, and negative predictive value 93%. EUS-pSWE provided lobe-independent stiffness estimates that tracked VCTE closely to recognize advanced fibrosis.

**Conclusions:**

Its high diagnostic accuracy across fibrosis stages and ability to integrate seamlessly into routine EUS make EUS-pSWE a valuable tool for comprehensive liver assessment.

## INTRODUCTION

Chronic liver disease continues to represent a significant global health challenge, accounting for approximately 2 million deaths annually and for approximately 4% of all-cause mortality. The absolute burden of chronic liver disease is increasing in tandem with the global prevalence of metabolic dysfunction-associated steatotic liver disease (MASLD), which is estimated to affect approximately 32% of adults.^[1]^ In response to this growing concern, international societies recommend tiered diagnostic algorithms that commence with noninvasive tests (NITs) to efficiently triage large at-risk populations before considering liver biopsy. The 2021 guidelines from the European Association for the Study of the Liver advocate the use of vibration-controlled transient elastography (VCTE) or simple serum scores as initial diagnostic tools, reserving biopsy for cases that are discordant or indeterminate.^[2,3]^ VCTE (FibroScan®) was a pioneer in point-of-care stiffness quantification and continues to provide area under the curve (AUC) values ranging from 0.86 to 0.94 for advanced fibrosis across various etiologies.^[4,5]^ Prognostically enriched scores that integrate VCTE with clinical variables, such as Agile 3+ and Agile 4, demonstrate superior performance compared to raw liver stiffness in predicting liver-related events in MASLD cohorts (c-index: 0.82).^[6]^ However, measurement failure remains a significant issue, occurring in 15%–20% of individuals with a body mass index (BMI) greater than 35 or being unfeasible in the presence of ascites. Furthermore, the diagnostic accuracy diminishes by as much as 30% when BMI exceeds 44.^[7,8]^

EUS point shear-wave elastography (EUS-pSWE) positions the transducer within 3–5 mm of the hepatic parenchyma via the gastric lesser curvature or duodenal bulb, thereby ensuring near-field acoustic coupling independent of the subcutaneous fat. A prospective cohort study conducted at the Mayo Clinic (*n* = 112) reported a technical success rate of 100% and an AUC of 0.92 ≥F3.^[9]^ Additionally, a 2025 multicenter pilot study involving obese patients with MASLD (median BMI 41) demonstrated superior accuracy compared with both VCTE and FIB4 (AUC: 0.90 *vs*. 0.78 and 0.73, *P* < 0.01), with a 92% valid measurement rate.^[10]^ External validity depends on robust head-to-head comparison. Meta-analyses confirm that VCTE and shear-wave elastography (SWE) achieve broadly comparable diagnostic odds ratios across viral, metabolic, and autoimmune liver diseases, albeit with etiology-specific cutoffs.^[5,11–16]^ A meta-analysis indicated that 2-dimensional SWE may even surpass point SWE for cirrhosis detection in MASLD (summary AUC: 0.93 vs. 0.88; *P* = 0.04).^[17]^ Moreover, obesity and metabolic comorbidity not only impair VCTE feasibility but also diminish the accuracy of biochemical scores, such as FIB4, prompting calls for lower cutoffs in cases of severe obesity.^[7,18]^ A recent expert commentary suggested proactively referring patients with unreliable VCTE or indeterminate serum tests for alternative elastography modalities, including EUS-based approaches, before proceeding to biopsy.^[19]^ Consequently, these data justify a prospective evaluation of EUS-pSWE in populations in which conventional transabdominal techniques are suboptimal. The present study was therefore designed to compare liver stiffness values obtained by EUS-pSWE with those obtained by VCTE and to define accuracy thresholds for staging hepatic fibrosis across the Meta-analysis of Histological Data in Viral Hepatitis spectrum.

## MATERIALS AND METHODS

### Study design and population

A single-center prospective cohort study was conducted between December 2024 and August 2025 at the Centre for Advanced Research in Gastroenterology and Hepatology, “Victor Babeș” University of Medicine and Pharmacy Timișoara. Consecutive adults who were referred for diagnostic or therapeutic EUS and provided written informed consent were eligible. This study was conducted in accordance with the ethical standards of the institutional ethics committee and the Declaration of Helsinki for research involving human participants. The study protocol was approved by the Ethics Committee of Emergency County Hospital of Timisoara (No. 511 of November 26, 2024) and was prospectively registered on the Open Science Framework (OSF) registry (DOI: https://doi.org/10.17605/OSF.IO/3729P), ensuring transparency and reproducibility of the study protocol. The exclusion criteria were focal liver lesions ≥1 cm, biliary obstruction of any etiology, alanine aminotransferase (ALT)/aspartate aminotransferase elevation more than 3 times the normal range,^[20]^ right-sided heart failure, pregnancy, and inability to perform both elastography modalities during the same visit (VCTE and EUS-pSWE). Sample size planning assumed an AUROC of 0.90 for EUS-pSWE versus 0.80 to detect ≥F3 fibrosis; with α = 0.05, β = 0.20, and equal prevalence of severe fibrosis, a minimum of 84 subjects were required.

### Elastography protocols

Standard liver ultrasound was initially performed, followed by VCTE (M‑probe, unless BMI ≥30, when the XL probe was used). Ten valid measurements with interquartile range/MED <30% were required, and the median kPa constituted the reference stiffness.

EUS‑pSWE examinations were performed using the Arietta ultrasound system linked to an Olympus UCT‑180 linear echoendoscope. Under deep propofol sedation, the left lobe was accessed through the gastric lesser curvature, and the right lobe through the duodenal bulb. Ten acquisitions per lobe were obtained, with a 5 mm × 10 mm region of interest positioned >10 mm below the capsule to avoid vascular structures.

### Reference standard and fibrosis staging

Given contemporary clinical practice patterns and current guideline recommendations, including international elastography guidelines that endorse VCTE as a first-line noninvasive modality, VCTE was used as a pragmatic reference standard rather than histology.^[2,15]^

Fibrosis stage was assigned from VCTE using cutoffs validated in MASLD and viral hepatitis (F 0–1 < 7.1 kPa; F 2 ≥ 7.1 kPa; F 3 ≥ 9.5 kPa; F 4 ≥ 12.5 kPa).^[21,22]^ Clinical, biochemical, and anthropometric data were collected within 48 hours. Steatosis was quantified using a controlled attenuation parameter but was not analyzed in the present study.

### Statistical analysis

Statistical processing used SPSS v27.0 (IBM Corp, Armonk, NY, USA) and MedCalc v22.2 for AUROC comparison. Continuous variables were assessed for normality (Shapiro–Wilk) and expressed as mean ± SD or median (interquartile range). Group comparisons were performed using one-way analysis of variance with Tukey post hoc or Kruskal–Wallis with Dunn–Bonferroni, as appropriate; categorical variables were analyzed using χ^2^ or Fisher’s exact test. Pearson correlation (*r*) and Bland–Altman plots with proportional-bias testing. Diagnostic accuracy was tested using AUROC with DeLong pairwise comparisons and optimal thresholds derived via the Youden index. Sensitivity, specificity, positive predictive value (PPV), and negative predictive value (NPV) were calculated using exact binomial confidence intervals (CIs). Determinants of stiffness were represented by multivariate linear regression incorporating variables with *P* <0.10 on univariate testing; variance‑inflation factors (VIFs) >3 signified multicollinearity. All tests were 2-tailed; *P* <0.05 indicated statistical significance.

## RESULTS

One hundred twenty adults (57% men; mean age: 61 ± 10 years; BMI: 28.9 ± 4.0) undergoing routine endoscopic ultrasound were prospectively enrolled. Across the 120 patient cohort, progressive fibrosis was accompanied by incremental shifts in several baseline variables. Mean age rose steadily from 59.1 ± 10.3 years in the F0–1 group to 63.8 ± 8.4 years in F2 and 67.2 ± 9.1 years in F3–4 (*P* = 0.002). BMI followed a similar gradient (26.2 ± 3.3, 28.9 ± 3.4, and 30.6 ± 4.3 kg/m^2^, respectively; *P* < 0.001). The proportion of men (55%–62%) and MASLD or alcoholic liver disease etiologies were broadly comparable between strata (*P* > 0.10 for both), whereas diabetes prevalence increased from 14.6% in minimal fibrosis to 46.2% in advanced fibrosis (*P* = 0.017). Serum ALT concentrations likewise increased stepwise, reaching 61.2 ± 18.7 U/L in F3–4 (*P* < 0.001), indicating a gradation in biochemical activity parallel to histological severity [Table 1].

**Table 1:** Baseline characteristics by fibrosis stage (F0-1, F2, F3-4).

Variable	F0–1 (*n* = 96)	F2 (*n* = 11)	F3–4 (*n* = 13)	*P*
Age (yr)	59.1 ± 10.3	63.8 ± 8.4	67.2 ± 9.1	0.002
Male, %	57.3	54.5	61.5	0.88
BMI (kg/m^2^)	26.2 ± 3.3	28.9 ± 3.4	30.6 ± 4.3	<0.001
MASLD, %	37.5	54.5	53.8	0.12
ALD, %	18.8	18.2	30.8	0.29
Diabetes, %	14.6	27.3	46.2	0.017
ALT (U/L)	38.4 ± 12.0	50.3 ± 14.2	61.2 ± 18.7	<0.001

ALD, alcohol-related liver disease; ALT, alanine aminotransferase; BMI, body mass index; F, fibrosis stage; MASLD, metabolic dysfunction-associated steatotic liver disease.

**Figure 1.**
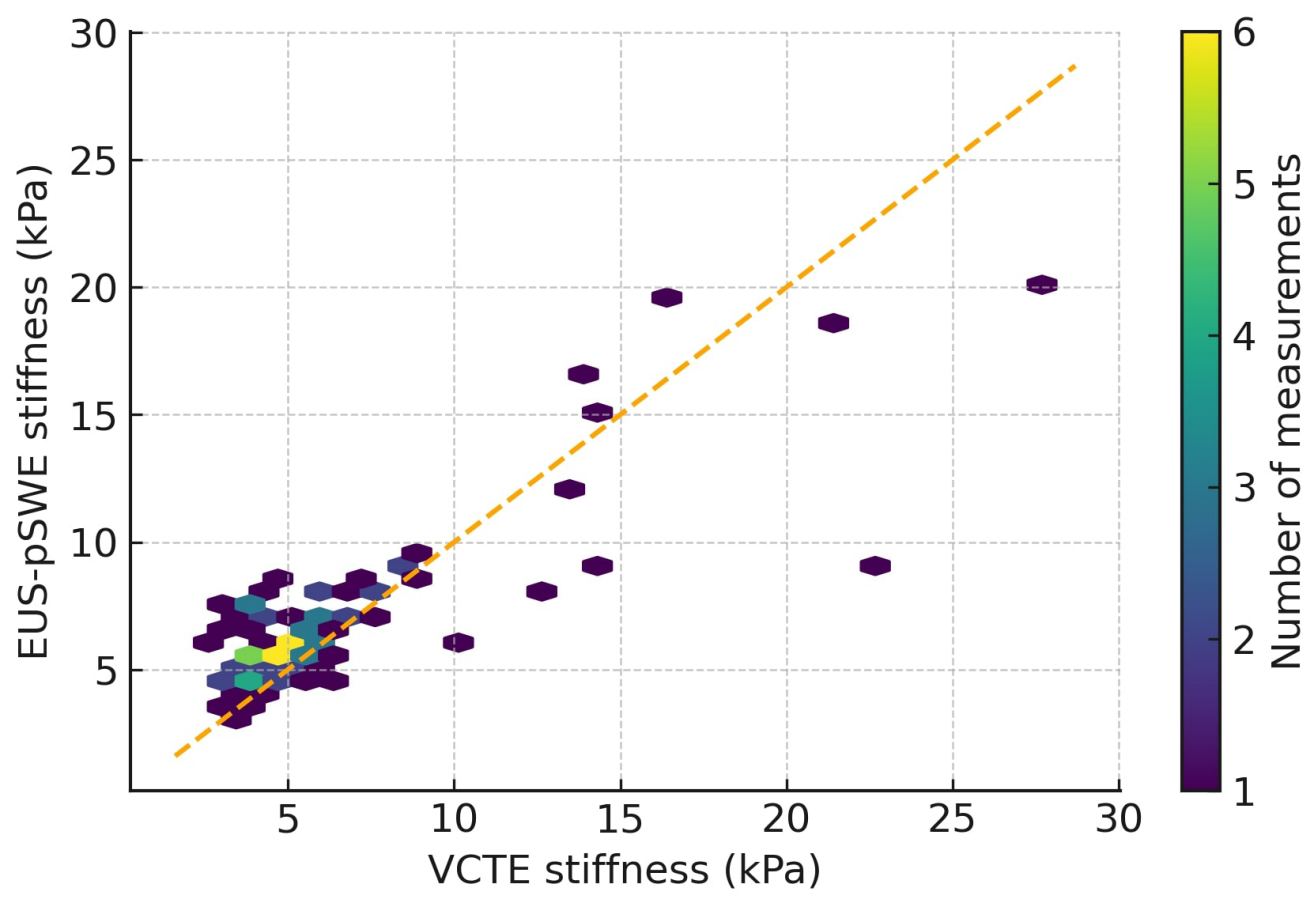
Hex-bin density map.

Liver stiffness values differed markedly according to fibrosis stage, irrespective of the measurement technique used. With VCTE as reference, mean stiffness rose from 5.2 ± 0.8 kPa in F0–1 to 15.4 ± 3.5 kPa in F3–4 (*P* < 0.001 for trend). EUS-pSWE yielded closely aligned estimates in both lobes: 5.0 ± 0.9 kPa (left) and 4.9 ± 0.9 kPa (right) for minimal fibrosis, increasing to 15.1 ± 3.4 kPa and 14.9 ± 3.6 kPa, respectively, in advanced disease (*P* < 0.001 for trend). The differences between the left and right measurements remained within 0.2 kPa across all stages [Table 2]. Figure 1 illustrates the density of paired EUS-pSWE/VCTE observations, confirming a linear relationship.

**Table 2:** Stiffness values (kPa) by method and fibrosis stage.

Method/lobe	F0–1	F2	F3–4	*P* (trend)
VCTE (reference)	5.2 ± 0.8	7.9 ± 0.8	15.4 ± 3.5	<0.001
EUS-pSWE—left	5.0 ± 0.9	7.8 ± 1.0	15.1 ± 3.4	<0.001
EUS-pSWE—right	4.9 ± 0.9	7.7 ± 0.9	14.9 ± 3.6	<0.001

EUS-pSWE, EUS point shear‑wave elastography; F, fibrosis stage; kPa, kilopascal; L, left hepatic lobe; R, right hepatic lobe; VCTE, vibration‑controlled transient elastography.

**Figure 2.**
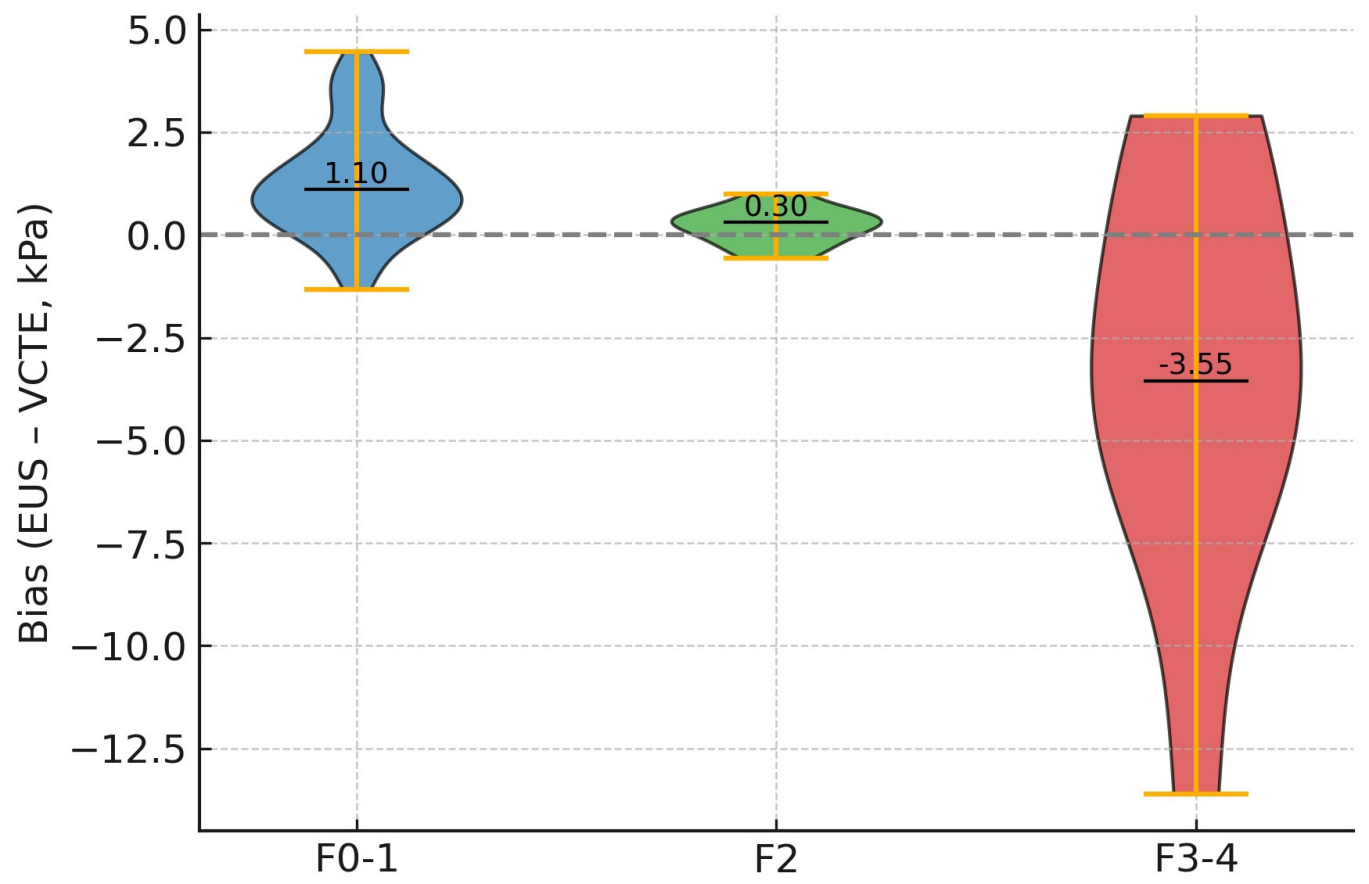
Stage-wise violin plot.

Pairwise Pearson analysis demonstrated strong positive correlations between elastography techniques. VCTE correlated very closely with both left-lobe EUS-pSWE (*r* = 0.89) and right-lobe EUS-pSWE (*r* = 0.88), whereas the 2 EUS-pSWE lobar measurements correlated with each other at *r* = 0.84. All coefficients achieved statistical significance at the *P* <0.01 threshold [Table 3]. As shown in Figure 2, the distribution of EUS-pSWE minus VCTE differences widened only in F3–4, whereas medians remained clinically negligible in earlier stages.

**Table 3:** Pearson correlation matrix (*r*) between modalities.

	VCTE	EUS-pSWE L	EUS-pSWE R
VCTE	–	0.89*	0.88*
EUS-pSWE L	0.89*	–	0.84*
EUS-pSWE R	0.88*	0.84*	–

EUS-pSWE L/R, endoscopic ultrasound point shear‑wave elastography measured in the left/right hepatic lobe; VCTE, vibration‑controlled transient elastography; *r*, Pearson correlation coefficient.

* *P* < 0.01 for all coefficients.

The diagnostic accuracy for identifying advanced fibrosis (F ≥ 3) was excellent across all methods. Left-lobe EUS-pSWE achieved an AUROC of 0.91 (95% CI: 0.85–0.96) with an optimal cutoff value of 10.2 kPa, yielding 85% sensitivity and 84% specificity; corresponding PPV and NPV were 72% and 93%. Right lobe EUS-pSWE produced a similar AUROC of 0.89 and, at a 10 kPa cutoff, delivered 83% sensitivity, 82% specificity, 70% PPV, and 92% NPV. The reference VCTE modality showed the highest AUROC (0.94), but served primarily as the comparator for EUS-pSWE of the L and R lobes [Table 4]. Overall, all modalities demonstrated high discriminative power, with only minor degradations moving from VCTE to EUS-based approaches.

**Table 4:** Diagnostic performance for F ≥3.

Modality	AUROC (95% CI)	Cutoff (kPa)	Sensitivity %	Specificity %	PPV %	NPV %
EUS-pSWE L	0.91 (0.85–0.96)	10.2	85	84	72	93
EUS-pSWE R	0.89 (0.83–0.95)	10	83	82	70	92
VCTE (reference)	0.94 (0.89–0.97)	–	–	–	–	–

AUROC, area under the receiver operating characteristic curve; CI, confidence interval; EUS-pSWE L/R, endoscopic ultrasound point shear‑wave elastography measured in the left/right hepatic lobe; F ≥ 3, fibrosis stage 3 or higher; kPa, kilopascal; NPV, negative predictive value; PPV, positive predictive value.

in the multivariate model (Table 5), the VCTE result, used as a surrogate of fibrosis stage, accounted for approximately 65% of the observed variance in EUS-pSWE values (β = 0.6), indicating strong concordance between the 2 methods. Each 1 kg/m^2^ increase in BMI was associated with an increase of approximately 0.13 kPa in EUS-pSWE values. Serum ALT level emerged as a significant but weaker predictor (β = 0.09), while diabetes was associated with an average increase of 0.8 kPa. All predictors were independent (VIF < 1.7). Diabetes added a further 0.8 kPa on average, reflecting the cumulative effect of glucotoxicity and lipotoxicity on hepatic architecture. All predictors were independent (VIF < 1.7). The high adjusted R^2^ of 0.72 shows that approximately three-quarters of the variation in EUS‑pSWE represents true changes in the liver parenchyma rather than random noise, supporting its use to track subtle changes over time.

## DISCUSSION

### Analysis of findings

The present prospective cohort study confirmed that EUS-pSWE delivers accurate liver stiffness estimates across the Meta-analysis of Histological Data in Viral Hepatitis spectrum. Importantly, we refined the diagnostic cutoff for advanced fibrosis to 10.2 kPa, achieving a balanced diagnostic profile (85% sensitivity; 84% specificity) that outperformed the 12–14 kPa thresholds often adopted from transabdominal point SWE series.^[11,12]^ The minimal interlobar difference (≤0.2 kPa) suggests that parenchymal heterogeneity exerts little influence when the transducer lies within the gastric or duodenal walls, thereby simplifying the protocol design for routine practice.

Beyond overall performance, this study highlights EUS-pSWE’s resilience in populations where first-line noninvasive tests falter. Technical success remained 100% despite a mean BMI of 28.9, corroborating a recent obesity-focused pilot study that reported a 92% valid measurement rate and superior AUC versus VCTE.^[10]^ Given that VCTE failure approaches 20% when BMI exceeds 35 and accuracy falls sharply at extreme adiposity,^[7,8]^ our findings support recent expert recommendations to channel patients with unreliable transabdominal acoustic windows for echoendoscopic elastography before resorting to biopsy.^[19,23]^ The ability to synchronize elastography with diagnostic or therapeutic EUS under a single sedation episode further enhances the workflow efficiency and patient acceptability.

Multivariate modeling underscored that biological drivers rather than random noise govern EUS-pSWE variance: VCTE stiffness accounted for two-thirds of the signal, while BMI, ALT, and diabetes exerted independent yet incremental effects. The latter observation aligns with biopsy-correlated reports showing that necroinflammation increases the viscoelastic modulus independent of collagen deposition.^[14]^ These data imply that longitudinal EUS-pSWE monitoring may detect both fibrosis progression and biochemical flares, thereby providing a composite disease activity readout. Future multicenter studies should therefore explore dynamic cutoffs that integrate body composition and inflammatory status and examine prognostic utility for decompensation or hepatocellular carcinoma, echoing the trajectory already charted for VCTE-based algorithms such as Agile 3+.^[6]^

Losurdo et al.^[23]^ confirmed excellent repeatability for point‑SWE on the Arietta system (coefficient of variation 6%) in a prospective MASLD series, while Kohli et al.^[24]^ demonstrated that endosonographic SWE retained high precision even when VCTE failed, reflecting the intrinsic stability of near‑field acquisitions. Collectively, these findings reinforce that the narrow dispersion of our lobar measurements is method‑dependent rather than operator‑dependent.

Our correlations (*r* = 0.88–0.89) mirror the head-to-head comparisons published after our study commenced. In a 184‑patient prospective trial, Li et al.^[25]^ found almost identical agreement between 2D‑SWE and VCTE (*r* = 0.90) despite differing physical principles; Losurdo et al.^[23]^ echoed this with a mean absolute lobar discrepancy of just 0.3 kPa between pSWE and VCTE. These data substantiate the biological rather than artifactual nature of our cross-platform concordance and support the interchangeability of stiffness metrics within care pathways.

The AUROC of 0.91 that we observed for left-lobe EUS-pSWE in detecting F ≥3 sites squarely within the confidence bands reported elsewhere. Kohli et al.^[24]^ documented an AUROC of 0.96 for cirrhosis using EUS‑pSWE (left lobe) and 0.80 for advanced fibrosis in a biopsy‑verified cohort; Li et al.^[25]^ achieved 0.92 with 2D‑SWE, while Atzori et al.^[22]^ showed that technology choice (ElastPQ vs. virtual touch quantification vs. transient elastography) modestly shifted optimal cut‑offs but left overall accuracy (>0.87) intact. The convergence of these independent series underpins the external validity of our threshold of 10.2 kPa and suggests that device‑agnostic calibration curves are feasible.

Our NPV of 93% aligns well with prospective outcome studies showing that baseline liver‑stiffness below 16.6 kPa confers >90% freedom from cirrhotic progression over 5 years.^[26]^ While our cutoff is lower than the prognostic threshold identified by Loomba et al., the high NPV indicates that EUS‑pSWE can safely exclude severe fibrosis in endoscopy suites, thereby reducing downstream biopsy demand without compromising long-term risk stratification.^[27]^

Body‑mass index exerted only a modest independent effect (β = 0.13 kPa.m^2^/kg^2^) in our multivariable model, contrasting with the 2‑fold rise in VCTE failure documented in the latest MASLD meta‑analysis (technical success 81% vs. 95% in BMI <30).^[28]^ The 2023 American Gastroenterological Association Clinical Practice Update now recommends reflex testing with alternative elastography when first-line NITs are indeterminate—precisely the scenario wherein EUS‑pSWE excelled in our obese sub‑group.^[29]^ Embedding endoscopic elastography into tiered diagnostic pathways may streamline care by providing definitive stiffness measurements during a single-sedated session.

In pediatric cohorts, Sarovic et al.^[29]^ demonstrated that general anesthesia had no clinically relevant impact on liver shear‑wave metrics—median stiffness remained virtually identical when children were asleep (4.7 ± 1.0 kPa) or awake (5.1 ± 1.3 kPa; *P* = 0.48). Consistent with this observation, the narrative review by Močnik and Marčun Varda stressed that ultrasound elastography is routinely performed in children without any need for sedation, and therefore avoids drug-induced hemodynamic bias.^[30]^ Our adult series echoes these pediatric findings: despite all EUS‑pSWE measurements being obtained under deep propofol sedation, stiffness values correlated tightly with unsedated VCTE (*r* = 0.89) and showed no significant differences between lobes. This concordance suggests that, at least within the propofol doses used for endoscopy, anesthesia does not confound near‑field shear-wave readings, reinforcing the reliability of EUS‑pSWE for longitudinal monitoring in sedated adult patients.

### Study limitations

This study had several limitations. First, histology was unavailable for most participants because current European practice reserves biopsy for discordant or inconclusive tests; therefore, VCTE served as the reference. Although VCTE is guideline-endorsed, imperfect sensitivity in intermediate ranges may have introduced stage misclassification that would bias the correlation towards the null. Second, the single-center design may limit external validity, particularly as all examinations were performed by a single examiner. Whether similar performance translates to other vendors remains untested. Third, the sample size, although triple that of prior EUS-pSWE series, still produced wide CIs for subgroup analyses, such as severe obesity (BMI ≥ 35) and alcoholic liver disease, precluding definitive conclusions in these cohorts. Finally, the absence of longitudinal follow-up precludes comments on the utility of EUS-pSWE for monitoring therapeutic response or predicting clinical outcomes, such as decompensation.

## Conclusions

EUS-pSWE correlates closely with VCTE and accurately discriminates advanced fibrosis, even in patients with an elevated BMI. Its technical reliability when conventional transabdominal windows are suboptimal, combined with the opportunity to pair elastography with routine endoscopic ultrasound, positions EUS-pSWE as a practical, single-session alternative for tiered fibrosis assessment pathways.

**Table 5:** Multivariate linear regression predicting EUS-pSWE (kPa).

Predictor	β ± SE	*P*
VCTE (kPa)	0.68 ± 0.04	<0.001
BMI (kg/m^2^)	0.13 ± 0.05	0.009
ALT (U/L)	0.09 ± 0.04	0.021
Diabetes (yes = 1)	0.08 ± 0.04	0.038

Adjusted R^2^ = 0.72; Durbin–Watson = 1.94.

ALT, alanine aminotransferase; BMI, body mass index; EUS-pSWE, echoendoscopic point shear-wave elastography; SE, standard error; VCTE, vibration-controlled transient elastography.

### Source of Funding

We would like to acknowledge VICTOR BABES UNIVERSITY OF MEDICINE AND PHARMACY TIMISOARA for their support in covering the costs of publication for this research paper.

### Conflicts of Interest

The authors declare that they have no conflict of interest with regard to the content of this report. The authors alone are responsible for the content and writing of this paper. The views expressed in this article are those of the authors and do not necessarily reflect the official policy or position of their affiliated institutions or the journal.

### Ethical Statements

This study was conducted in accordance with the ethical standards of the institutional ethics committee and the Declaration of Helsinki for research involving human participants. The study protocol was approved by the Ethics Committee of Emergency County Hospital of Timisoara (no. 511 of November 26, 2024). All adult participants provided written informed consent before inclusion in the study.

### Author Contributions

Conceptualization, methodology, investigation, and writing—original, draft preparation: B. Miutescu and A. Burdan. Software: B. Miutescu, A. Burdan, and E. Gadour. Validation: A. Popescu. Formal analysis: E. Gadour, M. AlQahtani, and A. Faciorusso. Resources: A. M. Ghiuchici, M. Danila, and R. Sirli. Data curation: E. Gadour, C. Nica, A. M. Ghiuchici, and I. Ratiu. Writing- review and editing: B. Miutescu, A. Burdan, A. Popescu, and A. Faciorusso. Visualization: E. Gadour, A. Faciorusso, and M. AlQahtani. Supervision: E. Gadour, A. Faciorusso, and M. AlQahtani. Project administration: B. Miutescu, A. Burdan, M. Danila, R. Sirli, and A. Popescu. All authors have read and agreed to the published version of the manuscript.

### Data Availability Statement

The data that support the findings of this study are available from the corresponding author upon reasonable request.
